# Role of CC Chemokines Subfamily in the Platinum Drugs Resistance Promotion in Cancer

**DOI:** 10.3389/fimmu.2020.00901

**Published:** 2020-05-15

**Authors:** Maria E. Reyes, Marjorie de La Fuente, Marcela Hermoso, Carmen G. Ili, Priscilla Brebi

**Affiliations:** ^1^Laboratorio de Biología Integrativa (LIBi), Centro de Excelencia en Medicina Traslacional-Scientific and Technological Bioresource Nucleus (CEMT-BIOREN), Universidad de la Frontera, Temuco, Chile; ^2^Laboratorio de Inmunidad Innata, Programa de Inmunología, ICBM, Facultad de Medicina, Universidad de Chile, Santiago, Chile; ^3^Dirección Académica, Clínica Las Condes, Santiago, Chile

**Keywords:** inflammation, cancer, CC chemokine subfamily, platinum drugs, chemoresistance

## Abstract

Cancer is a significant medical issue, being one of the main causes of mortality around the world. The therapies for this pathology depend on the stage in which the cancer is found, but it is usually diagnosed at an advanced stage in which the treatment is chemotherapy. Platinum drugs are among the most commonly used in therapy, unfortunately, one of the main obstacles to this treatment is the development of chemoresistance, which is the ability of cancer cells to evade the effects of drugs. Although some molecular mechanisms involved in resistance to platinum drugs are described, elucidation is still required of others. Secretion of inflammatory mediators such as cytokines and chemokines, by tumor microenvironment components or tumor cells, show direct influence on proliferation, metastasis and progression of cancer and are related to chemoresistance and poor prognosis. In this review, the general mechanisms associated with resistance to platinum drugs, inflammation on cancer development and chemoresistance in various types of cancer will be approached with special emphasis on the current history of CC chemokines subfamily-mediated chemoresistance.

## Introduction

Cancer is a major public health problem, one of the leading causes of death around the world (1). Therapies depend on the stage in which the cancer is found, but usually, diagnosis is at an advanced stage, in which treatment choice is chemotherapy. However, cancer cells develop chemoresistance through a combination of cellular and molecular mechanisms, and are consequently related to poor prognosis and lower patient's survival (2). Inflammation mediated by cytokines and chemokines has been linked to cancer initiation, promotion, and chemoresistance (3), all associated with the tumor microenvironment (TME) composed of stem cells, cancer cells, endothelial cells, immune cells, as well as fibroblasts and the extracellular matrix (ECM) (4). As part of the TME, cancer-associated fibroblasts (CAF) are involved in tumor progression, metastasis and drug resistance. CAFs are activated fibroblasts triggering signals involved in growth, differentiation and therapy evasion, and also secrete growth factors (epidermal growth factor, EGF) and IL-6 cytokine (4). Also, cytokines and chemokines are secreted by the TME and cancer cells through paracrine/autocrine mechanisms associated with chemoresistance (5). In the present review, the general mechanisms associated with inflammation in cancer development and platinum drug chemoresistance, and in particular, the role of CC chemokines subfamily in chemoresistance will be discussed.

## Cancer and Platinum Drugs

Cancer is a major cause of mortality worldwide (1), with cancer therapy depending on the tumor stage, which unfortunately, as with most cancers is diagnosed in stages in which the tumor is spread, with low survival rates (6). When chemotherapy is needed in advanced cases of cancer, one of the most used drugs is the platinum-based (6), although a major problem in cancer treatment, in addition to late diagnosis, is chemoresistance.

Platinum drugs are widely used in the treatment of different tumors, three of these compounds are approved by the United States Food and Drug Administration: cisplatin, carboplatin and oxaliplatin (7). Platinum drugs enter the cell through diffusion or by plasma membrane-mediated transporters (CTR1), usually allowing copper influx (8), by binding to methionine, histidine or cysteine CTR1 residues (9) to act as enzyme cofactor (10). Once inside the cell, platinum drugs bind to proteins, reduced Glutathione (GSH), and DNA N-7 site of purines (11). Adduct blocks DNA transcription and synthesis, and DNA repair mechanisms, triggering cell cycle arrest and apoptosis (12). Carboplatin forms a greater amount of intra-strand adducts compared to cisplatin, but the formation rate is 10 times slower, related to a lower toxicity (13). Compared to cisplatin, oxaliplatin induces potentially more lethal functional lesions, with greater cytotoxicity in human tumor cell lines, requiring less DNA lesions than cisplatin to inhibit cell growth (13).

Platinum drugs are an effective way to treat cancer, however, drug resistance may hinder therapy (14). Resistance to platinum drugs could develop through several mechanisms: decreased drug entry into the cell, increased expulsion (8, 11), increased detoxification (15, 16), increased DNA repair pathways (12, 17), upregulation of anti-apoptotic proteins such as Bcl-2, Bcl-XL, MCL-1(11), among others. Alternatively, epithelial mesenchymal transition (EMT) accompanies the development of drug resistance, with several molecules associated with EMT, such as transcription factors (Snail, Twist) and miRNAs (miRNA-200 family, miR-15, miR-186, etc.), being recognized as important for drug resistance (18) with effect in diverse signaling pathways associated with epithelial–mesenchymal transition such as STAT3, Notch, SMAD (19). In addition, DNA methylation of tumor suppressor genes and histone modifications are important resistance mechanisms (20). Finally, recent investigations associate epigenetic regulations as potential resistance mechanisms (21), with cisplatin resistance regulated by microRNAs and methylation/demethylation of genes such as FANCF in ovarian cancer, and related to cytokines/chemokines (axis CXCL12-CXCR4) (8) to be studied in detail later in the review. Examples of reported tumors developing resistance to cisplatin are ovarian cancer (22, 23) usually developed during treatment (acquired resistance) (24), cervical (25, 26), lung (27, 28), and gastric cancer (29, 30); the last two can also develop intrinsic resistance, occurring when the drug is ineffective from the beginning of treatment (14, 31).

## Inflammation and Cancer

Inflammation is a physiological response to cell damage by injury or infection (32), with pathogens not only related to chronic inflammation, but also immune system deregulation or autoimmunity, such as inflammatory bowel diseases, which increases colon cancer risk (33). Currently, about 20% of malignant tumors are related to chronic inflammation, including colon, gastric, liver, breast and lung cancer (34), with this phenomenon first observed in 1863 by Rudolph Virchow describing tumor leukocytes (35). Subsequently, the role of inflammatory cells described “chemical mediators” in the development of an inflammatory condition (36), currently known as cytokines and chemokines. Cytokines are low molecular weight polypeptide/glycoproteins synthesized by immune cells, stromal cells (fibroblasts and endothelial cells) (37) and tumor cells. Cytokines are responsible of proliferation, cell survival, differentiation, immune cell activation, cell migration, and death. Chemokines are a group of secreted proteins within the cytokine family of early induction (20), being a group of small proteins (8–12 kDa) stimulating lymphocyte migration from blood to tissues (chemotaxis), inducing integrin expression (38, 39). Cytokines and chemokines act in an autocrine manner, being endogenously synthesized by cells, and when they are secreted act on the same producing cell through specific receptors. In paracrine regulation, chemokines are produced and secreted by a cell acting in adjacent cells, sensed through specific receptors (40). The most studied cytokines in cancer are: tumor necrosis factor (TNF-α), involved in angiogenesis and invasion; Interleukin-1 (IL-1), associated with metastasis, and IL-8-associated proliferation and migration (41). Therefore, inflammation is involved at various stages of tumor development: in initiation favoring mutation development and increasing reactive oxygen and nitrogen species causing DNA damage (3). Meanwhile, immune cells infiltrating the tumor produce cytokines, activating key transcription factors (NF-κB, STAT3, and AP-1), and participate in tumor progression and angiogenesis. In summation, the main signaling pathways involved in the relationship of inflammation and cancer are NF-κB, STAT3, PI3K/Akt, and MAPK (35).

## Cellular Components From the Tumor Microenvironment (TME)

In recent years, the concept of TME has been introduced, consisting of various cells including cancer, mesenchymal, endothelial, immune, together with ECM, and fibroblasts contributing to tumor progression (4). Tumors are more complex than just a set of malignant cancer cells, since tumor cells efficiently recruit immune and vascular cells through secretion of growth factors, chemokines and cytokines. These recruited cells release growth-promoting signals and intermediate metabolites, allowing tissue structure remodeling, and reciprocal communication between cancer cells and TME eventually leads to increased proliferation and metastatic capacity (42).

## Cancer Associated Fibroblasts (CAF)

The fibroblasts present in the TME “activated” through TGF-β (released from tumor cells) generate CAF, with particular characteristics differentiating it from non-activated fibroblasts: star shape, expression of alpha smooth muscle actin (α-SMA) and fibroblast activation protein (FAP) markers (43). In addition to secretory phenotype, CAFs reshape the ECM and autocrine/dynamic activation in immune signaling functions, allowing persistent stimulus for tumor development favoring growth of tumor cells and metastasis (44). CAFs remodel tumor vasculature through secretion of VEGF, FGF and IL-6, and ECM, through secretion of matrix metalloproteinases (MMPs) and ECM proteins. Furthermore, they modulate pro-tumorigenic inflammation through secretion of IL-1, IL-6, TNF-α, TGF-β, and CCL2, favoring tumor growth, angiogenesis, invasion and metastasis (44, 45). In gastric cancer, CAFs also influence carcinogenesis through IL-6 induction in metastasis and invasion through factor overexpression increasing the epithelial-mesenchymal transition (EMT), finally activating the JAK2/STAT3 pathway (46). Additionally, the fibroblast growth factor-9 (FGF-9) secreted by CAFs trigger EMT and metastasis, together with CXCL12 and interleukin-11 inducing migration and invasion (46). In the case of inflammation associated with *Helicobacter pylori* infection, CAFs contribute to neoplastic transformation through activating a positive feedback mechanism of STX3-dependent COX-2, influencing STAT3 regulation via IL-6. Finally, induction of NF-κB increases cytidine deaminase expression leading to multiple mutations in the host genome such as those found in TP53 (47).

## Tumor-Associated Macrophages (TAMS)

TAMs refer to macrophages infiltrating the tumor and are not a homogenous cell population, but rather highly heterogenic cells participating in carcinogenesis (48). Usually, two extreme states of differentiation in macrophages are recognized: the classic phenotype (M1), associated to antitumor and pro-inflammatory activity [mediated by the secretion of cytokines IL-1β, TNF-α, and IL-6 (49)] and the alternative phenotype (M2), with pro-tumor and anti-inflammatory activity. M2 acts directly on the tumor cells and indirectly on the TME (50) by producing growth factors (Fibroblast Growth Factors, FGF; Vascular Endothelial Growth Factor, VEGF, and IL-6), matrix degrading enzymes and cytokines, thus inducing the neo-angiogenesis switch, tumor progression (37), tissue invasion and repair (51–54).

In colorectal cancer (CRC), TAMs show a greater infiltration in patients with better prognosis, or in those with less recurrence or complications (55–58), and are associated with a higher survival (59). Alternatively, M2-type macrophages are associated with a worse prognosis, less survival and later stages of disease (60, 61). TAMs with M2 profile produce enzymes and inhibitors regulating digestion of the ECM, metastasis and angiogenesis (62, 63) and additionally, control ECM composition directly or through the activation of fibroblasts, thus promoting tumor progression (64).

## Mesenchymal Stromal Cell (MSC)

MSC are adult multipotent stem cells located as pericytes in organs and tissues differentiating into specialized cells. Actually, MSC promote tumorigenic processes, such as angiogenesis, malignant cell, metastasis and chemoresistance (65). TME can be influenced by MSC through cytokine secretion and TGF-β involved in the EMT of carcinoma cells, necessary in favoring cancer progression (66). Alternatively, TNF-α-activated MSC promotes metastasis in lung cancer, through CCL5 and CCR2 ligands. Moreover, CXCR2 ligands (CXCL1, 2, and 5) induced by TNF-α-activated MSC recruit CXCR2^+^ neutrophils into tumor, responsible for the pro-metastatic effect of MSC (67).

## Cytokines and Chemoresistance

Cytokines have direct influence on cancer progression (5), secreted by both the TME and cancer cells, with TME cytokines inducing chemoresistance through paracrine regulation on tumor cells, promoting apoptosis inhibition, increased cell proliferation or drug efflux (5). In breast cancer, IL-6 and IL-8 are increased in resistant cells compared to parental cells sensitive to tamoxifen (5). Additionally, cisplatin-treated CAF increases IL-11 secretion, promoting drug resistance of lung adenocarcinoma through IL-11R/STAT3 pathway activation and subsequently upregulation of anti-apoptotic proteins (68). CAFs also secrete IL-11 promoting chemoresistance in gastric cancer through JAK/STAT3/Bcl-2 signaling pathway activation (69). Alternatively, cytokine three signaling suppressor (SOCS3), a negative cytokine regulator inhibiting the JAK/STAT pathway, is decreased in cisplatin-resistant lung tumor cells (70). Autocrine IL-6 or IL-8 secretion by ovarian cancer cells induces resistance to paclitaxel and cisplatin, due to decreased proteolytic caspase 3 activation, increased Bcl-2 expression, and MAPK and PI3K/Akt pathway activation (71, 72). In colon cancer, IL-17 and IL-6-mediated chemoresistance regulates Akt and STAT3 signaling pathways, respectively (73, 74). Lastly, in gastric cancer, CAFs secrete IL-6 inducing resistance to 5-fluorouracil or cisplatin, with inhibition of its receptor (IL-6R), suppressing drug resistance (46).

## CC Chemokines Subfamily and Chemoresistance

Chemokines coordinate leukocyte recruitment to tissues in physiological and pathological conditions, also mediating cell differentiation, proliferation and survival (75). Chemokines are a large subfamily of cytokines subdivided into 4 main classes (depending on location of the first two cysteine residues, C, in the protein sequence), such as: CC (first 2 adjacent cysteines), CXC (cysteines separated by another amino acid), C (cysteine in the amino terminal region), and CX3C (with three intermediate residues separating the cysteine). There is redundancy in this superfamily, with several ligands binding to the same receptors and vice versa (76). Chemokines act through G-protein coupled receptors, having 7 transmembrane regions, interacting with proteoglycan glycosamino-glycans, with a nomenclature associated with the binding-chemokine type: receptors for CC (CCR), CXC (CXCR), C (XCR1), and for CX3C (CX3CR1) (77). Chemokine binding triggers phosphorylation of serine/threonine residues in the receptor, this activation involves GTP binding to the Ga subunit of the Gb dissociation complex and initiating signaling pathways (PI3K, MAPK, and Rho) involved in proliferation, motility, and expression of MMPs and cytokines. Chemokine receptors also activate independent G protein pathways such as JAK/STAT regulating migration and gene transcription (78). The relationship of chemokines with tumor development can be indirect or direct. Indirect action acknowledges that tumors secrete chemokines attracting leukocytes producing growth factors, as CCL2 secretion increases M2-type TAM in breast cancer (79). Additionally, the tumor stroma may deliver inflammatory chemokines affecting tumor development, stroma-derived CXCL12 binds CXCR4 receptor possibly promoting tumor progression by stimulating angiogenesis (80). Direct chemokine action has been related to functional receptor expression by the tumor cells, with receptors associated with increased proliferation and survival. For example, CXCR4 receptor is expressed in tumors such as ovary, glioma, melanoma and renal, CXCR6 in prostate cancer, CXCR2 in melanoma and CCR6 in colorectal and pancreatic cancer (75). Moreover, CCL2 induces a pro-tumorigenic mechanism based on autocrine secretion and activation of CCR2, thus inhibiting apoptosis (80). CXC chemokine receptors have prognostic value in gastric cancer, with CXCR2 (81) and CXCR4 (82) related to poor prognosis, while CXCR3 has good prognosis (83). Regarding CC chemokines, CCL7 and CCL21 overexpression is associated with poor prognosis (84), CCR7 and CCR5 receptors associate with invasion and metastasis in gastric cancer, and lastly CCR7 is associated with EMT (85).

CC subfamily chemokines have been implicated in chemoresistance (Table 1). CCL5 activates STAT3 through an autocrine loop inhibiting caspase-9/PARP and modulates Bcl-2 (5). Autocrine regulation evades drug response, where tumor-derived cytokines activate signaling pathways involved in survival and proliferation, counteracting the effects of chemotherapy. In lung cancer, CCL2 is linked to Docetaxel resistance through PI3K/Akt pathway activation, inhibiting caspase 3-dependent apoptosis (101); this review is focused in platinum drugs, but chemokines also affect other cancer drugs such as Docetaxel (101) or Tamoxifen (102). Additionally in gastric cancer, CCL2 initiates chemoresistance to platinum drugs through PI3K/Akt/mTOR signaling pathway activation by inhibiting pro-apoptotic autophagy and increasing SQSTM1 (receptor member for autophagy) expression (86). In ovarian cancer, cisplatin induces CAF-derived CCL5 secretion, promoting drug resistance, mediated by PI3K and STAT3 signaling pathway regulation, inhibiting apoptosis and promoting proliferation (92). Also, stroma-derived CCL2/CCL5 induces IL-6 release from the tumor cell generating carboplatin resistance through PYK2 pathway activation (positioned upstream of the JAK1/STAT3 pathway), a critical mediator of survival pathway activation (91). Likewise, CCL20 is associated with doxorubicin resistance through MDR1 membrane transporter expression regulation (103). Finally, CCR9 receptor is associated with cisplatin resistance in ovarian (100) and breast cancer (99) through the PI3K pathway activation.

**Table 1:** Summary of chemokines of the CC sub-family in chemo resistance to platinum drugs.

**CC chemokine**	**Tumor**	**Pathway**	**Model**	**Mechanism**	**References**
CCL2	Gastric	PI3K	Resistant and sensitive co-culture	Decreases pro-apototic autophagy and increases SQSTM1	(86)
CCL2	Ovarian	–	*In vitro* and *in vivo*	EMT characteristic	(87)
CCL2	Lung	P38	Resistant v/s sensitive	P53 mediated apoptosis regulation	(88)
CCL2	Lung	NF-kB	*In vivo* tumor	LUBAC activation	(89)
CCL2/CCL4	Leukemia	NF-kB	Stromal mesenchymal cells	ARC (apoptosis repressor with caspase recruitment domain) /IL1β/ Mesenchymal	(90)
CCL2/CCL5	Ovarian	PYK2	Ascites (mesenchymal) and sensitive	Increase survival	(91)
CCL5	Ovarian	STAT3-PI3K	CAF	Decrease apoptosis and increase anti-apoptotic protein (bcl2)	(92)
CCL11	Ovarian	STAT3 y MAPK	Normal epithelium/tumor cell	Apoptosis control	(93)
CCL14/CCL15	Liver	PKC	Primary culture of human hepatocyte and human hepatoma cell line Alexander	The nuclear receptor (FXR) is involved in the regulation of CCL14 and CCL15. Loss of pro-apoptotic balance/survival.	(94)
CCL18	Lung	GPR30	Cell line A549	Regulation by epithelial-mesenchymal transition	(95)
CCL21	Ovarian	–	Bioinformatic analysis	–	(96)
CCL21	Lung	ERK	Cell lines A549 and H460	Overexpression of anti-apoptotic bcl-2 protein and decrease in pro-apoptotic proteins such as bax and caspase-3	(97)
CCL22	Lung	Src/CD155/MIF	Co-culture Macrophages with cell line	M2 polarization of TAM through MIF secretion	(98)
CCL25	Breast	PI3K	Two breast cancer cell lines	Activates cell survival signals and inhibits apoptosis	(99)
CCL25	Ovarian	PI3K	Cell lines OVCAR-3 y SKOV-3	Increased survival by phosphorylating and inactivating pro-apoptotic factors, such as FKHR and GSK-3β	(100)

## TME and CC Chemokine Mechanism to Induce Chemoresistance

**Figure 1:**
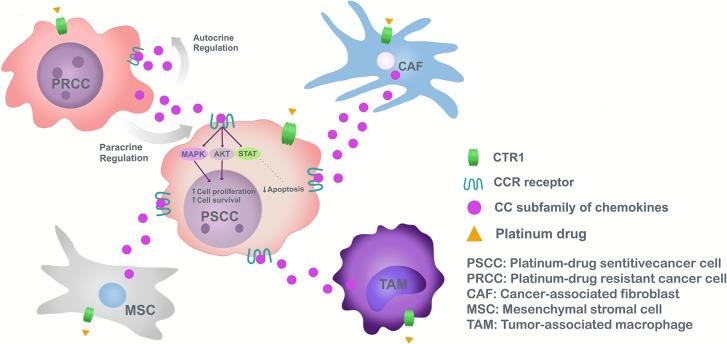
Model of chemoresistance induction to platinum drugs, mediated by CC subfamily chemokines in cancer.

Chemokines of the CC subfamily responsible for chemoresistance have different origins according to TME and tumor heterogeneity. In fact, chemokines are secreted in a paracrine manner from the TME mainly by macrophages, CAFs, or MSC (5) (Figure 1).

Monocytes/macrophages are recruited to the tumor area by cancer cell derived-CCL2 (104). Once connected the tumor, TAMs respond to external signals involving innate and adaptive immune processes (105). TAMs polarized to M2 in lung cancer through CCL22 (98), produce an immune suppressive effect, decreasing antitumor activity, spreading tumor cells and chemoresistance (106).

We previously demonstrated that CCL4 tumor levels have a positive correlation with the M2 marker (CD163) (107) associated with a poor clinical outcome of colorectal cancer and lower cumulative survival than patients with low CD163 expression (58).

In the case of CAFs, stimulation with cisplatin increases CCL5 secretion contributing to chemoresistance in ovarian cancer (92). CAF constitute a heterogeneous cell population, express a wide range of molecular markers (α-SMA, FAP), not necessarily exclusive to fibroblasts (108, 109), and may be related to cells undergoing EMT (110). The complexity of CAFs has been underestimated, with subsets tending to promote carcinogenesis (determined by type and location of tumor), have different markers from those generally used, and separating them according to origin: vCAF of perivascular cells, mCAF of resident fibroblasts and dCAF of tumor cells performing EMT (108).

Another TME component is MSC which protect ovarian cancer cells from carboplatin-induced apoptosis through inhibition of caspase activation, however, secrete high levels of IL-6 and IL-8 contributing to chemoresistance in breast cancer (105). MSC pre-treated with cisplatin increased CCL5 expression and phosphorylation of tyrosine kinases (PLC, WNK1, c-Jun, STAT3), possibly playing roles in tumor cell changes, as witnessed in breast cancer cells (111).

Due to the TME as a paracrine secretor of CC type chemokines in response to platinum drugs, chemokine production by tumor cells is studied. In this particular case, because of tumor heterogeneity and CAF origins, possibly resistant cells originating in the tumor secrete CC chemokines and induce chemoresistance in adjacent tumor cells (102). Resistant cells found in the heterogeneous population of cancer cells (due to aberrant DNA repair mechanisms and cell death pathway deregulation) have stem cell properties, forming tumors in distant organs contributing to pathology reappearance after a successful therapy (112). Cancer stem-like cells have received increasing attention, their existence observed in various tumor types, renewing themselves and differentiating into other cells. Also, cancer stem-like cells influence macrophage polarization (113), in addition to EMT, hindering development of better therapies to reduce cancer relapse (114). Tumor cells release autocrine chemokines aiding cisplatin chemoresistance by inactivating pro-apoptotic autophagy, as demonstrated by CCL2 in gastric cancer cell lines (86). This suggests that CCL2 not only maintain chemoresistance in drug-resistant tumoral cells but also confer drug resistance to drug-sensitive cancer cells (86) (Figure 1).

## CC Chemokine as Therapeutic Target

A current cancer treatment is the immunotherapy, and its principle based in the mechanism of T-cell–mediated immunity is a complicated succession of occasions, with continuous exchange among stimulatory and inhibitory signals, when T cells are active, dynamic dealt to explicit destinations by following a chemokine gradient and advancing cytotoxicity and tumor cell killing (115).

The main targets in cancer immunotherapy are the immune checkpoint receptor called programmed cell death protein 1 (PD-1) and cytotoxic T-lymphocyte Antigen 4 (CTLA-4) (116) two negative regulators of T-cell function. Inhibition of these targets resulting in increased activation of the immune system anti-tumor, has led to immunotherapies applied to treatment of melanoma, non–small cell lung cancer, and other cancers (117). Chemokines help with T cell trafficking, but some CC chemokine with pro-tumorigenic and drug-resistant function limit immunotherapy treatment.

Due to the above, the chemokines and chemokine receptors are a potential target to therapy and could reverses chemoresistance or synergizing with monotherapy of immune-treatment (116). Direct CC chemokine antibodies targeting is an option but the main target are CC chemokine receptor such as CCR1, CCR2, CCR4, CCR5, and CCR7 by monoclonal antibodies inhibitors or antagonism molecules (118) for example, Maraviroc is a CCR5 receptor antagonism that decrease metastasis in breast (119) and gastric cancer (120) and provoke remission in pancreatic and liver cancer by apoptosis induction (20). Receptor inhibition could influence in cancer pathway signaling (121) and interfere with autocrine chemokine synthesis (5, 91).

## Conclusion

TME cellular components (CAF, TAM, MSC) influence the secretion of CC subfamily chemokines in a paracrine manner inducing tumor progression, metastasis and platinum drug chemoresistance. Additionally, drug-resistant cancer cells can also secrete chemokines to the adjacent environment. Therefore, tumor cellular heterogeneity, cancer-resistant cells (cancer stem-like cells or cells in EMT), and particularly the TME components, are capable of producing and secreting CC chemokines inducing a resistant phenotype in adjacent cancer-sensitive cell.

## Author Contributions

MR wrote the first draft of the manuscript. MR, MF, and CI wrote sections of the manuscript. PB and MH made substantial contributions and discussed the content. All authors reviewed and/or edited the manuscript prior submission.

## Conflict of Interest

The authors declare that the research was conducted in the absence of any commercial or financial relationships that could be construed as a potential conflict of interest.

## References

[1] Bray F Ferlay J Soerjomataram I Siegel RL Torre LA Jemal A. Global cancer statistics 2018: GLOBOCAN estimates of incidence and mortality worldwide for 36 cancers in 185 countries. CA Cancer J Clin. (2018) 68:394–424. 10.3322/caac.21492 30207593

[2] Marin JJG Al-Abdulla R Lozano E Briz O Bujanda L Banales J. Mechanisms of Resistance to Chemotherapy in Gastric Cancer. Anticancer Agents Med Chem. (2016) 16:318–34. 10.2174/1871520615666150803125121 26234359

[3] Grivennikov SI Greten FR Karin M. Immunity, Inflammation, and Cancer. Cell. (2010) 140:883–99. 10.1016/j.cell.2010.01.025 20303878 PMC2866629

[4] Senthebane DA Rowe A Thomford NE Shipanga H Munro D Al Mazeedi MAM. The role of tumor microenvironment in chemoresistance: to survive, keep your enemies closer. Int J Mol Sci. (2017) 18:1586. 10.3390/ijms18071586 28754000 PMC5536073

[5] Jones VS Huang RYRP Chen LP Chen ZS Fu L. Cytokines in cancer drug resistance: cues to new therapeutic strategies. Biochim Biophys Acta. (2016) 1865:255–65. 10.1016/j.bbcan.2016.03.005 26993403

[6] Orditura M Galizia G Sforza V Gambardella V Fabozzi A Laterza MM. Treatment of gastric cancer. World J Gastroenterol. (2014) 20:1635–49. 10.3748/wjg.v20.i7.1635 24587643 PMC3930964

[7] Hato S V Khong A De Vries IJM Lesterhuis WJ. Molecular pathways: the immunogenic effects of platinum-based chemotherapeutics. Clin Cancer Res. (2014) 20:2831–7. 10.1158/1078-0432.CCR-13-3141 24879823

[8] Jain A Jahagirdar D Nilendu P Sharma NK. Molecular approaches to potentiate cisplatin responsiveness in carcinoma therapeutics. Expert Rev Anticancer Ther. (2017) 17:815–25. 10.1080/14737140.2017.1356231 28705091

[9] Öhrvik H Thiele DJ. How copper traverses cellular membranes through the mammalian copper transporter 1, Ctr1. Ann N Y Acad Sci. (2015) 1314:32–41. 10.1111/nyas.12371 24697869 PMC4158275

[10] Eisses JF Kaplan JH. Molecular characterization of hCTR1, the human copper uptake protein. J Biol Chem. (2002) 277:29162–71. 10.1074/jbc.M203652200 12034741

[11] Galluzzi L Senovilla L Vitale I Michels J Martins I Kepp O. Molecular mechanisms of cisplatin resistance. Oncogene. (2012) 31:1869–83. 10.1038/onc.2011.384 21892204

[12] Rocha C Silva M Quinet A Cabral-Neto J Menck C. DNA repair pathways and cisplatin resistance: an intimate relationship. Clinics. (2018) 73:1–10. 10.6061/clinics/2018/e478s 30208165 PMC6113849

[13] Fong CW. Platinum anti-cancer drugs: free radical mechanism of Pt-DNA adduct formation and anti-neoplastic effect. Free Radic Biol Med. (2016) 95:216–29. 10.1016/j.freeradbiomed.2016.03.006 27012421

[14] Florea A-MM Büsselberg D. Cisplatin as an anti-tumor drug: cellular mechanisms of activity, drug resistance and induced side effects. Cancers (Basel). (2011) 3:1351–71. 10.3390/cancers3011351 24212665 PMC3756417

[15] Stordal B Davey M. Understanding cisplatin resistance using cellular models. IUBMB Life. (2007) 59:696–9. 10.1080/15216540701636287 17885832

[16] De Luca A Parker LJ Ang WH Rodolfo C Gabbarini V Hancock NC. A structure-based mechanism of cisplatin resistance mediated by glutathione transferase P1-1. Proc Natl Acad Sci USA. (2019) 116:201903297. 10.1073/pnas.1903297116 31221747 PMC6628828

[17] Kartalou M Essigmann JM. Mechanisms of resistance to cisplatin. Mutat Res. - Fundam Mol Mech Mutagen. (2001) 478:23–43. 10.1016/S0027-5107(01)00141-5 11406167

[18] Brozovic A. The relationship between platinum drug resistance and epithelial–mesenchymal transition. Arch Toxicol. (2017) 91:605–19. 10.1007/s00204-016-1912-7 28032148

[19] Du B Shim JS. Targeting Epithelial–Mesenchymal Transition (EMT) to overcome drug resistance in cancer. Molecules. (2016) 21:1–15 10.3390/molecules21070965 27455225 PMC6273543

[20] Schwarzenbach H Gahan PB Chen H Cong X Wu C Wu XX Chemokines and chemokine receptors: new targets for cancer immunotherapy. Cancer Drug Resist. (2019) 10:1–10. 10.3389/fimmu.2019.00379

[21] Shen D-W Pouliot LM Hall MD Gottesman MM. Cisplatin resistance: a cellular self-defense mechanism resulting from multiple epigenetic and genetic changes. Pharmacol Rev. (2012) 64:706–21. 10.1124/pr.111.005637 22659329 PMC3400836

[22] Stronach EA Cunnea P Turner C Guney T Aiyappa R Jeyapalan S. The role of interleukin-8 (IL-8) and IL-8 receptors in platinum response in high grade serous ovarian carcinoma. Oncotarget. (2015) 6:31593–603. 10.18632/oncotarget.3415 26267317 PMC4741626

[23] Cohen S Bruchim I Graiver D Evron Z Oron-Karni V Pasmanik-Chor M. Platinum-resistance in ovarian cancer cells is mediated by IL-6 secretion via the increased expression of its target cIAP-2. J Mol Med. (2013) 91:357–68. 10.1007/s00109-012-0946-4 23052480

[24] Christie EL Bowtell DDL Acquired chemotherapy resistance in ovarian cancer. Ann Oncol. (2017) 28:2017–9. 10.1093/annonc/mdx446 29232469

[25] Wu S Li W Wu Z Cheng T Wang P Li N. TNFAIP8 promotes cisplatin resistance in cervical carcinoma cells by inhibiting cellular apoptosis. Oncol Lett. (2019) 17:4667–74. 10.3892/ol.2019.10076 30944654 PMC6444441

[26] Li R Liu GZ Luo SY Chen R Zhang JX. Cyclin i promotes cisplatin resistance via Cdk5 activation in cervical cancer. Eur Rev Med Pharmacol Sci. (2015) 19:4533–41. 26698249

[27] Xiao L Lan X Shi X Zhao K Wang D Wang X. Cytoplasmic RAP1 mediates cisplatin resistance of non-small cell lung cancer. Cell Death Dis. (2017) 8:e2803. 10.1038/cddis.2017.210 28518145 PMC5520727

[28] Cui Y Li G Zhang X Dai F Zhang R. Increased MALAT1 expression contributes to cisplatin resistance in non-small cell lung cancer. Oncol Lett. (2018) 16:4821–8. 10.3892/ol.2018.9293 30250547 PMC6144744

[29] Zhi X Tao J Xiang G Cao H Liu Z Yang K. APRIL induces cisplatin resistance in gastric cancer cells via activation of the NF-κB pathway. Cell Physiol Biochem. (2015) 35:571–85. 10.1159/000369720 25612651

[30] Huang D Duan H Huang H Tong X Han Y Ru G. Cisplatin resistance in gastric cancer cells is associated with HER2 upregulation-induced epithelial-mesenchymal transition. Sci Rep. (2016) 6:1–12. 10.1038/srep20502 26846307 PMC4742832

[31] Zeng-rong N Paterson J Alpert L Tsao M Jean Â Alaoui-jamali MA. Elevated DNA repair capacity is associated with intrinsic resistance of lung cancer to chemotherapy. Cancer Res. (1995) 55:4760–4. 7585500

[32] Shalapour S Karin M Shalapour S Karin M. Immunity, inflammation, and cancer : an eternal fight between good and evil. J Clin Invest. (2015) 125:3347–55. 10.1172/JCI80007 26325032 PMC4588298

[33] Axelrad JE Lichtiger S Yajnik V. Inflammatory bowel disease and cancer: the role of inflammation, immunosuppression, and cancer treatment. World J Gastroenterol. (2016) 22:4794–801. 10.3748/wjg.v22.i20.4794 27239106 PMC4873872

[34] Galdiero MR Marone G Mantovani A. Cancer Inflammation and Cytokines. Cold Spring Harb Perspect Biol. (2018) 10:1–18. 10.1101/cshperspect.a028662 28778871 PMC6071493

[35] Multhoff G Molls M Radons J. Chronic Inflammation in Cancer Development. Front Immunol. (2012) 2:1–17. 10.3389/fimmu.2011.00098 22566887 PMC3342348

[36] Korniluk A Koper O Kemona H Dymicka-Piekarska V. From inflammation to cancer. Ir J Med Sci. (2017) 186:57–62. 10.1007/s11845-016-1464-0 27156054 PMC5323483

[37] Landskron G De La Fuente M Thuwajit P Thuwajit C Hermoso MA. Chronic inflammation and cytokines in the tumor microenvironment. J Immunol Res. (2014) 2014, 10.1155/2014/149185 24901008 PMC4036716

[38] Turner MD Nedjai B Hurst T Pennington DJ. cytokines and chemokines: at the crossroads of cell signalling and inflammatory disease. Biochim Biophys Acta - Mol Cell Res. (2014) 1843:2563–82. 10.1016/j.bbamcr.2014.05.014 24892271

[39] Ferreira VL Borba HHL Bonetti AF Leonart LP Pontarolo R Cytokines and interferons: types and functions. In: Ali Khan W editor. Autoantibodies and Cytokines. IntechOpen (2018). 10.5772/intechopen.74550

[40] Lázár-Molnár E Hegyesi H Tóth S Falus A. Autocrine and paracrine regulation by cytokines and growth factors in melanoma. Cytokine. (2000) 12:547–54. 10.1006/cyto.1999.0614 10843728

[41] Balkwill F Mantovani A. Inflammation and cancer: back to Virchow? Lancet (London, England). (2001) 357:539–45. 10.1016/S0140-6736(00)04046-0 11229684

[42] Yuan Y Jiang YC Sun CK Chen QM. Role of the tumor microenvironment in tumor progression and the clinical applications (Review). Oncol Rep. (2016) 35:2499–515. 10.3892/or.2016.4660 26986034

[43] Kalluri R Zeisberg M. Fibroblasts in cancer. Nat Rev Cancer. (2006) 6:392–401. 10.1038/nrc1877 16572188

[44] Qu X Tang Y Hua S. Immunological approaches towards cancer and inflammation: a cross talk. Front Immunol. (2018) 9:1–19. 10.3389/fimmu.2018.00563 29662489 PMC5890100

[45] LeBleu VS Kalluri R. A peek into cancer-associated fibroblasts: origins, functions and translational impact. Dis Model Mech. (2018) 11:1–9. 10.1242/dmm.029447 29686035 PMC5963854

[46] Ham I-H Lee D Hur H. Role of cancer-associated fibroblast in gastric cancer progression and resistance to treatments. J Oncol. (2019) 2019:1–11. 10.1155/2019/6270784 31281359 PMC6590541

[47] Ida S Watanabe M Baba H Chronic inflammation and gastrointestinal cancer. J Cancer Metastasis Treat. (2015) 1:138–43. 10.4103/2394-4722.166994

[48] Mantovani A Locati M. Tumor-associated macrophages as a paradigm of macrophage plasticity, diversity, and polarization lessons and open questions. Arterioscler Thromb Vasc Biol. (2013) 33:1478–83. 10.1161/ATVBAHA.113.300168 23766387

[49] Mantovani A Sica A Locati M. New vistas on macrophage differentiation and activation. Eur J Immunol. (2007) 37:14–6. 10.1002/eji.200636910 17183610

[50] Norton SE Ward-Hartstonge K Taylor ES Kemp R. Immune cell interplay in colorectal cancer prognosis. World J Gastrointest Oncol. (2015) 7:221–32. 10.4251/wjgo.v7.i10.221 26483876 PMC4606176

[51] Genin M Clement F Fattaccioli A Raes M Michiels C. M1 and M2 macrophages derived from THP-1 cells differentially modulate the response of cancer cells to etoposide. BMC Cancer. (2015) 15:577. 10.1186/s12885-015-1546-9 26253167 PMC4545815

[52] Comito G Giannoni E Segura CP Barcellos-de-Souza P Raspollini MR Baroni G. Cancer-associated fibroblasts and M2-polarized macrophages synergize during prostate carcinoma progression. Oncogene. (2014) 33:2423–31. 10.1038/onc.2013.191 23728338

[53] Gordon S Martinez FO. Alternative activation of macrophages: mechanism and functions. Immunity. (2010) 32:593–604. 10.1016/j.immuni.2010.05.007 20510870

[54] Erreni M Mantovani A Allavena P. Tumor-associated Macrophages (TAM) and inflammation in colorectal cancer. Cancer Microenviron. (2011) 4:141–54. 10.1007/s12307-010-0052-5 21909876 PMC3170420

[55] Nakayama Y Nagashima N Minagawa N Inoue Y Katsuki T Onitsuka K. Relationships between tumor-associated macrophages and clinicopathological factors in patients with colorectal cancer. Anticancer Res. (2002) 22:4291–6. 12553072

[56] Kinouchi M Miura K Mizoi T Ishida K Fujibuchi W Sasaki H. Infiltration of CD40-positive tumor-associated macrophages indicates a favorable prognosis in colorectal cancer patients. Hepatogastroenterology. (2013) 60:83–8. 10.5754/hge12372 22687258

[57] Bingle L Brown NJ Lewis CE. The role of tumour-associated macrophages in tumour progression: implications for new anticancer therapies. J Pathol. (2002) 196:254–65. 10.1002/path.1027 11857487

[58] Herrera M Herrera A Domínguez G Silva J García V García JM. Cancer-associated fibroblast and M2 macrophage markers together predict outcome in colorectal cancer patients. Cancer Sci. (2013) 104:437–44. 10.1111/cas.12096 23298232 PMC7657228

[59] Gulubova M Ananiev J Yovchev Y Julianov A Karashmalakov A Vlaykova T. The density of macrophages in colorectal cancer is inversely correlated to TGF-β1 expression and patients' survival. J Mol Histol. (2013) 44:679–92. 10.1007/s10735-013-9520-9 23801404

[60] Komohara Y Fujiwara Y Ohnishi K Takeya M. Tumor-associated macrophages: Potential therapeutic targets for anti-cancer therapy. Adv Drug Deliv Rev. (2016) 99:180–5. 10.1016/j.addr.2015.11.009 26621196

[61] Zhang Y Sime W Juhas M Sjölander A. Crosstalk between colon cancer cells and macrophages via inflammatory mediators and CD47 promotes tumour cell migration. Eur J Cancer. (2013) 49:3320–34. 10.1016/j.ejca.2013.06.005 23810249

[62] Mantovani A Sozzani S Locati M Allavena P Sica A. Macrophage polarization: tumor-associated macrophages as a paradigm for polarized M2 mononuclear phagocytes. Trends Immunol. (2002) 23:549–55. 10.1016/S1471-4906(02)02302-5 12401408

[63] Isidro R Appleyard CB. Colonic macrophage polarization in homeostasis, inflammation, and cancer. Am J Physiol. - Gastrointest Liver Physiol. (2016) 311:G59–73. 10.1152/ajpgi.00123.2016 27229123 PMC4967174

[64] Afik R Zigmond E Vugman M Klepfish M Shimshoni E Pasmanik-Chor M. Tumor macrophages are pivotal constructors of tumor collagenous matrix. J Exp Med. (2016) 213:2315–31. 10.1084/jem.20151193 27697834 PMC5068227

[65] Nwabo KAH Kamga PT Simo RT Vecchio L Seke EPF Muller JM. Mesenchymal stromal cells' role in tumor microenvironment: involvement of signaling pathways. Cancer Biol Med. (2017) 14:129. 10.20892/j.issn.2095-3941.2016.0033 28607804 PMC5444925

[66] Poggi A Varesano S Zocchi MR. How to hit mesenchymal stromal cells and make the tumor microenvironment immunostimulant rather than immunosuppressive. Front Immunol. (2018) 9:1–17. 10.3389/fimmu.2018.01342 29515580 PMC5825917

[67] Yu PF Huang Y Han YY Lin LY Sun WH Rabson AB. TNFα-activated mesenchymal stromal cells promote breast cancer metastasis by recruiting CXCR2+ neutrophils. Oncogene. (2017) 36:482–90. 10.1038/onc.2016.217 27375023 PMC5290040

[68] Tao L Huang G Wang R Pan Y He Z Chu X. Cancer-associated fibroblasts treated with cisplatin facilitates chemoresistance of lung adenocarcinoma through IL-11/IL-11R/STAT3 signaling pathway. Sci Rep. (2016) 6:38408. 10.1038/srep38408 27922075 PMC5138853

[69] Ma Y Zhu J Chen S Li T Ma J Guo S. Activated gastric cancer-associated fibroblasts contribute to the malignant phenotype and 5-FU resistance via paracrine action in gastric cancer. Cancer Cell Int. (2018) 18:1–12. 10.1186/s12935-018-0599-7 30038550 PMC6053778

[70] Xu Y Tang H Gong L Hu H Chen L Sui X Interferon-γ upregulates SOCS3 expression to reduce cisplatin chemoresistance in non-small cell lung cancer A549 cells. Chemother Open Access. (2016) 5:1–4. 10.4172/2167-7700.1000205

[71] Wang Y Niu XL Qu Y Wu J Zhu YQ Sun WJ. Autocrine production of interleukin-6 confers cisplatin and paclitaxel resistance in ovarian cancer cells. Cancer Lett. (2010) 295:110–23. 10.1016/j.canlet.2010.02.019 20236757

[72] Wang Y Niu XL Qu Y Wu J Zhu YQ Sun WJ. Autocrine production of interleukin-8 confers cisplatin and paclitaxel resistance in ovarian cancer cells. Cytokine. (2011) 56:365–75. 10.1016/j.cyto.2011.06.005 21742513

[73] Yin Y Yao S Hu Y Feng Y Li M Bian Z. The immune-microenvironment confers chemoresistance of colorectal cancer through macrophage-derived IL6. Clin Cancer Res. (2017) 23:7375–87. 10.1158/1078-0432.CCR-17-1283 28928161

[74] Sui G Qiu Y Yu H Kong Q Zhen B. Interleukin-17 promotes the development of cisplatin resistance in colorectal cancer. Oncol Lett. (2019) 17:944–50. 10.3892/ol.2018.9645 30655852 PMC6313016

[75] Bonecchi R Savino B Mantovani A Locati M Targeting Chemokines in Cancer. Curr Immunol Rev. (2012) 8:161–9. 10.2174/157339512800099648

[76] Nagarsheth N Wicha MS Zou W. Chemokines in the cancer microenvironment and their relevance in cancer immunotherapy. Nat Rev Immunol. (2017) 17:559–72. 10.1038/nri.2017.49 28555670 PMC5731833

[77] Dowsland M Harvey J Lennard T Kirby J Ali S. Chemokines and breast cancer: a gateway to revolutionary targeted cancer treatments? Curr Med Chem. (2005) 10:579–92. 10.2174/0929867033457944 12678790

[78] Hembruff SL Cheng N. Chemokine signaling in cancer: implications on the tumor microenvironment and therapeutic targeting. Cancer Ther. (2009) 7:254–67. 20651940 PMC2907742

[79] Li D Ji H Niu X Yin L Wang Y Gu Y. Tumor-associated macrophages secrete CCL2 and induce tamoxifen resistance by activating PI3K/Akt/mTOR in breast cancer. Cancer Sci. (2019) 111:47–58. 10.1111/cas.14230 31710162 PMC6942430

[80] Rollins BJ. Inflammatory chemokines in cancer growth and progression. Eur J Cancer. (2006) 42:760–7. 10.1016/j.ejca.2006.01.002 16510278

[81] Wang Z Liu H Shen Z Wang X Zhang H Qin J. The prognostic value of CXC-chemokine receptor 2 (CXCR2) in gastric cancer patients. BMC Cancer. (2015) 15:1–8. 10.1186/s12885-015-1793-9 26497045 PMC4619066

[82] He H Wang C Shen Z Fang Y Wang X Chen W. Upregulated Expression of C-X-C chemokine receptor 4 is an independent prognostic predictor for patients with gastric cancer. PLoS ONE. (2013) 8:1–7. 10.1371/journal.pone.0071864 23936528 PMC3735563

[83] Chen F Yuan J Yan H Liu H Yin S Duan B. Chemokine receptor CXCR3 correlates with decreased M2 macrophage infiltration and favorable prognosis in gastric cancer. Biomed Res Int. (2019) 2019:6832867. 10.1155/2019/6832867 31240220 PMC6556258

[84] Hwang TL Lee LY Wang CC Liang Y Huang SF Wu CM. CCL7 and CCL21 overexpression in gastric cancer is associated with lymph node metastasis and poor prognosis. World J Gastroenterol. (2012) 18:1249–56. 10.3748/wjg.v18.i11.1249 22468089 PMC3309915

[85] Ryu H Baek S Moon J Jo I Kim N Lee H. C-C motif chemokine receptors in gastric cancer (Review). Mol Clin Oncol. (2017) 8:3–8. 10.3892/mco.2017.1470 29285394 PMC5738695

[86] Xu W Wei Q Han M Zhou B Wang H Zhang J. CCL2-SQSTM1 positive feedback loop suppresses autophagy to promote chemoresistance in gastric cancer. Int J Biol Sci. (2018) 14:1054–66. 10.7150/ijbs.25349 29989092 PMC6036739

[87] Moisan F Francisco EB Brozovic A Duran GE Wang YC Chaturvedi S. Enhancement of paclitaxel and carboplatin therapies byCCL2 blockade in ovarian cancers. Mol Oncol. (2014) 8:1231–9. 10.1016/j.molonc.2014.03.016 24816187 PMC4801026

[88] Sarin N Engel F Kalayda G V Mannewitz M Cinatl J Rothweiler F. Cisplatin resistance in non-small cell lung cancer cells is associated with an abrogation of cisplatin-induced G2/M cell cycle arrest. PLoS ONE. (2017) 12:1–26. 10.1371/journal.pone.0181081 28746345 PMC5528889

[89] Ruiz EJ Diefenbacher ME Nelson JK Sancho R Pucci F Chakraborty A. LUBAC determines chemotherapy resistance in squamous cell lung cancer. J Exp Med. (2019) 216:450–65. 10.1084/jem.20180742 30642944 PMC6363428

[90] Carter BZ Mak PY Chen Y Mak DH Mu H Jacamo R. Anti-apoptotic ARC protein confers chemoresistance by controlling leukemia-microenvironment interactions through a NFκB/IL1β signaling network. Oncotarget. (2016) 7:20054–67. 10.18632/oncotarget.7911 26956049 PMC4991438

[91] Pasquier J Gosset M Geyl C Hoarau-véchot J Chevrot A Pocard M. CCL2/CCL5 secreted by the stroma induce IL-6/PYK2 dependent chemoresistance in ovarian cancer. Mol Cancer. (2018) 17:1–14. 10.1186/s12943-018-0787-z 29455640 PMC5817856

[92] Zhou B Sun C Li N Shan W Lu H Guo L. Cisplatin-induced CCL5 secretion from CAFs promotes cisplatin-resistance in ovarian cancer via regulation of the STAT3 and PI3K/Akt signaling pathways. Int J Oncol. (2016) 48:2087–97. 10.3892/ijo.2016.3442 26983899

[93] Levina V Nolen BM Marrangoni AM Cheng P Marks JR Szczepanski MJ. Role of eotaxin-1 signaling in ovarian cancer. Clin Cancer Res. (2009) 15:2647–56. 10.1158/1078-0432.CCR-08-2024 19351767 PMC2669845

[94] Vaquero J Briz O Herraez E Muntané J Marin JJG. Activation of the nuclear receptor FXR enhances hepatocyte chemoprotection and liver tumor chemoresistance against genotoxic compounds. Biochim Biophys Acta - Mol Cell Res. (2013) 1833:2212–9. 10.1016/j.bbamcr.2013.05.006 23680185

[95] Ploenes T Scholtes B Krohn A Burger M Passlick B Müller-Quernheim J. CC-Chemokine ligand 18 induces epithelial to mesenchymal transition in lung cancer A549 cells and elevates the invasive potential. PLoS ONE. (2013) 8:e53068. 10.1371/journal.pone.0053068 23349697 PMC3548837

[96] Yin F Liu X Li D Wang Q Zhang W Li L. Bioinformatic analysis of chemokine (C-C motif) ligand 21 and SPARC-like protein 1 revealing their associations with drug resistance in ovarian cancer. Int J Oncol. (2013) 42:1305–16. 10.3892/ijo.2013.1819 23404140

[97] Xu Y Liu L Qiu X Liu Z Li H Li Z. CCL21/CCR7 prevents apoptosis via the ERK pathway in human non-small cell lung cancer cells. PLoS ONE. (2012) 7:3–10. 10.1371/journal.pone.0033262 22438908 PMC3306387

[98] Huang WC Kuo KT Wang CH Yeh CT Wang Y. Cisplatin resistant lung cancer cells promoted M2 polarization of tumor-associated macrophages via the Src/CD155/MIF functional pathway. J Exp Clin Cancer Res. (2019) 38:1–17. 10.1186/s13046-019-1166-3 31036057 PMC6489343

[99] Johnson-Holiday C Singh R Johnson EL Grizzle WE Lillard JW Singh S. CCR9-CCL25 interactions promote cisplatin resistance in breast cancer cell through Akt activation in a PI3K-dependent and FAK-independent fashion. World J Surg Oncol. (2011) 9:1–7. 10.1186/1477-7819-9-46 21539750 PMC3110128

[100] Johnson EL Singh R Johnson-Holiday CM Grizzle WE Partridge EE Lillard JW. CCR9 interactions support ovarian cancer cell survival and resistance to cisplatin-induced apoptosis in a PI3K-dependent and FAK-independent fashion. J Ovarian Res. (2010) 3:1–8. 10.1186/1757-2215-3-15 20565782 PMC2914045

[101] Wang T Zhan Q Peng X Qiu Z Zhao T. CCL2 influences the sensitivity of lung cancer A549 cells to docetaxel. Oncol Lett. (2018) 16:1267–74. 10.3892/ol.2018.8769 30061946 PMC6063033

[102] Yi EH Lee CS Lee J Lee YJ Shin MK Cho C. STAT3-RANTES autocrine signaling is essential for tamoxifen resistance in human breast cancer cells. (2013) 11:31–43. 10.1158/1541-7786.MCR-12-0217 23074171

[103] Su S Sun X Zhang Q Zhang Z Chen J. CCL20 promotes ovarian cancer chemotherapy resistance by regulating ABCB1 expression. Cell Struct Funct. (2019) 44:21–8. 10.1247/csf.18029 30760665 PMC11926410

[104] Ireland L V Mielgo A. Macrophages and fibroblasts, key players in cancer chemoresistance. Front Cell Dev Biol. (2018) 6:1–14. 10.3389/fcell.2018.00131 30356656 PMC6189297

[105] Chen W Qin Y Liu S. Cytokines, breast cancer stem cells (BCSCs) and chemoresistance. Clin Transl Med. (2018) 7:1–13. 10.1186/s40169-018-0205-6 30175384 PMC6119679

[106] Solís-Martínez R Hernández-Flores G Ochoa-Carrillo FJ Ortiz-Lazareno P Bravo-Cuellar A Tumor-associated macrophages contribute to the progression of prostate cancer. Gac Mex Oncol. (2015) 14:97–102. 10.1016/j.gamo.2015.03.001

[107] De la Fuente López M Landskron G Parada D Dubois-Camacho K Simian D Martinez M. The relationship between chemokines CCL2, CCL3, and CCL4 with the tumor microenvironment and tumor-associated macrophage markers in colorectal cancer. Tumour Biol. (2018) 40:1–12. 10.1177/1010428318810059 30419802

[108] Gieniec KA Butler LM Worthley DL Woods SL. Cancer-associated fibroblasts—heroes or villains? Br J Cancer. (2019) 121:293–302. 10.1038/s41416-019-0509-3 31289350 PMC6738083

[109] Kalluri R. The biology and function of fibroblasts in cancer. Nat Rev Cancer. (2016) 16:582–98. 10.1038/nrc.2016.73 27550820

[110] Baulida J. Epithelial-to-mesenchymal transition transcription factors in cancer-associated fibroblasts. Mol Oncol. (2017) 11:847–59. 10.1002/1878-0261.12080 28544627 PMC5496490

[111] Skolekova S Matuskova M Bohac M Toro L Demkova L Gursky J. Cisplatin-induced mesenchymal stromal cells-mediated mechanism contributing to decreased antitumor effect in breast cancer cells. Cell Commun Signal. (2016) 14:1–13. 10.1186/s12964-016-0130-5 26759169 PMC4710002

[112] Housman G Byler S Heerboth S Lapinska K Longacre M Snyder N. Drug resistance in cancer: an overview. Cancers (Basel). (2014) 6:1769–92. 10.3390/cancers6031769 25198391 PMC4190567

[113] Zhang Q Cai DJ Li B. Ovarian cancer stem-like cells elicit the polarization of M2 macrophages. Mol Med Rep. (2015) 11:4685–93. 10.3892/mmr.2015.3323 25672286

[114] Singh A Settleman J. EMT, cancer stem cells and drug resistance : an emerging axis of evil in the war on cancer. Oncogene. (2010) 29:4741–51. 10.1038/onc.2010.215 20531305 PMC3176718

[115] Fares CM Allen EM Van Drake CG Allison JP Mechanisms of resistance to immune checkpoint blockade: why does checkpoint inhibitor immunotherapy not work for all patients? Am Soc Clin Oncol Educ Book. (2020) 39:147–64. 10.1200/EDBK_240837 31099674

[116] Vilgelm AE Richmond A. Chemokines modulate immune surveillance in tumorignesis, metastatsis, and response to immunotherapy. Front Immunol. (2019) 10:6–8. 10.3389/fimmu.2019.00333 30873179 PMC6400988

[117] Buchbinder EI Desai A. CTLA-4 and PD-1 pathways: similarities, differences, and implications of their inhibition. Am J Clin Oncol. (2016) 39:98–106. 10.1097/COC.0000000000000239 26558876 PMC4892769

[118] Poeta VM Massara M Capucetti A Bonecchi R. Chemokines and chemokine receptors: new targets for cancer immunotherapy. Front Immunol. (2019) 10:1–10. 10.3389/fimmu.2019.00379 30894861 PMC6414456

[119] Pervaiz A Zepp M Mahmood S Ali DM Berger MR. CCR5 blockage by maraviroc : a potential therapeutic option for metastatic breast cancer. Cell Oncol (Dordr). (2019) 42:93–106. 10.1007/s13402-018-0415-3 30456574 PMC12994360

[120] Mencarelli A Graziosi L Renga B Cipriani S D'Amore C Francisci D. CCR5 antagonism by maraviroc reduces the potential for gastric cancer cell dissemination. Transl Oncol. (2014) 6:784–93. 10.1593/tlo.13499 24466382 PMC3890714

[121] Aldinucci D Casagrande N. Inhibition of the CCL5/CCR5 axis against the progression of gastric cancer. Int J Mol Sci. (2018) 19:1477. 10.3390/ijms19051477 29772686 PMC5983686

